# Supplementary material for: Saccharomyces boulardii (CNCM I-745) improves intestinal damage in sepsis by remodeling intestinal flora structure
Source: Front Cell Infect Microbiol. 2025 Jul 31;15:1602792. doi: 10.3389/fcimb.2025.1602792 (PMC12350329; doi:10.3389/fcimb.2025.1602792)
Supplement: Supplementary file 1 [file DataSheet1.zip › summary/summary/8_Advanced_analyse/2_PICRUSt2_result/KEGG/Group.DW0_vs_BW0/PICRUSt2_KO_Group.DW0_vs_BW0_diff.pdf]

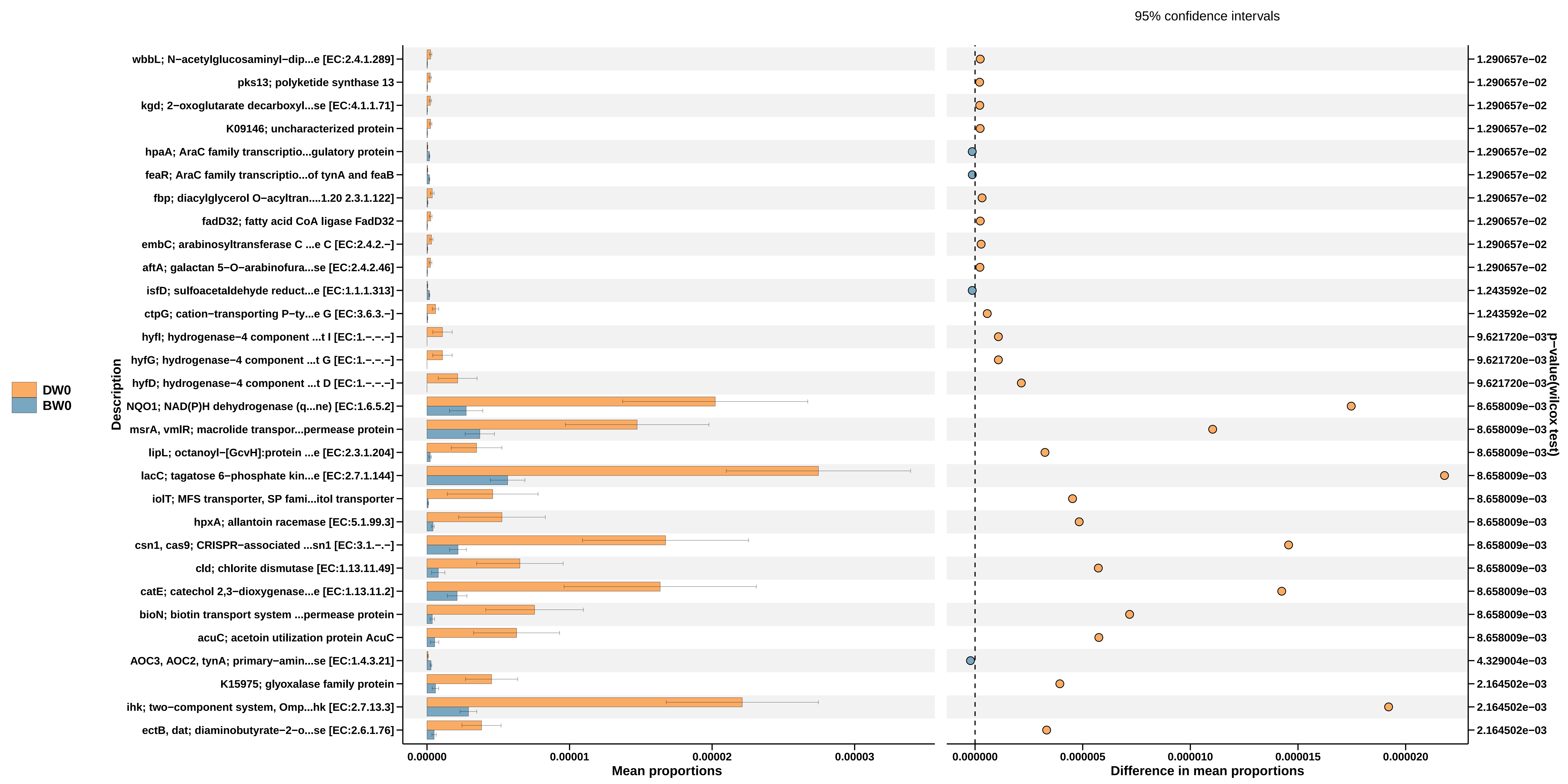
95% confidence intervals
DW0
BW0
Description
wbbL; N−acetylglucosaminyl−dip...e [EC:2.4.1.289]
pks13; polyketide synthase 13
kgd; 2−oxoglutarate decarboxyl...se [EC:4.1.1.71]
K09146; uncharacterized protein
hpaA; AraC family transcriptio...gulatory protein
feaR; AraC family transcriptio...of tynA and feaB
fbp; diacylglycerol O−acyltran....1.20 2.3.1.122]
fadD32; fatty acid CoA ligase FadD32
embC; arabinosyltransferase C ...e C [EC:2.4.2.−]
aftA; galactan 5−O−arabinofura...se [EC:2.4.2.46]
isfD; sulfoacetaldehyde reduct...e [EC:1.1.1.313]
ctpG; cation−transporting P−ty...e G [EC:3.6.3.−]
hyfI; hydrogenase−4 component ...t I [EC:1.−.−.−]
hyfG; hydrogenase−4 component ...t G [EC:1.−.−.−]
hyfD; hydrogenase−4 component ...t D [EC:1.−.−.−]
NQO1; NAD(P)H dehydrogenase (q...ne) [EC:1.6.5.2]
msrA, vmlR; macrolide transpor...permease protein
lipL; octanoyl−[GcvH]:protein ...e [EC:2.3.1.204]
lacC; tagatose 6−phosphate kin...e [EC:2.7.1.144]
iolT; MFS transporter, SP fami...itol transporter
hpxA; allantoin racemase [EC:5.1.99.3]
csn1, cas9; CRISPR−associated ...sn1 [EC:3.1.−.−]
cld; chlorite dismutase [EC:1.13.11.49]
catE; catechol 2,3−dioxygenase...e [EC:1.13.11.2]
bioN; biotin transport system ...permease protein
acuC; acetoin utilization protein AcuC
AOC3, AOC2, tynA; primary−amin...se [EC:1.4.3.21]
K15975; glyoxalase family protein
ihk; two−component system, Omp...hk [EC:2.7.13.3]
ectB, dat; diaminobutyrate−2−o...se [EC:2.6.1.76]
0.00000
0.00001
0.00002
0.00003
Mean proportions
0.000000
0.000005
0.000010
0.000015
0.000020
Difference in mean proportions
p−value(wilcox test)
1.290657e−02
1.290657e−02
1.290657e−02
1.290657e−02
1.290657e−02
1.290657e−02
1.290657e−02
1.290657e−02
1.290657e−02
1.290657e−02
1.243592e−02
1.243592e−02
9.621720e−03
9.621720e−03
9.621720e−03
8.658009e−03
8.658009e−03
8.658009e−03
8.658009e−03
8.658009e−03
8.658009e−03
8.658009e−03
8.658009e−03
8.658009e−03
8.658009e−03
8.658009e−03
4.329004e−03
2.164502e−03
2.164502e−03
2.164502e−03
